# Distinct Mechanism of Cysteine Oxidation-Dependent Activation and Cold Sensitization of Human Transient Receptor Potential Ankyrin 1 Channel by High and Low Oxaliplatin

**DOI:** 10.3389/fphys.2017.00878

**Published:** 2017-11-01

**Authors:** Takahito Miyake, Saki Nakamura, Zhao Meng, Satoshi Hamano, Keisuke Inoue, Tomohiro Numata, Nobuaki Takahashi, Kazuki Nagayasu, Hisashi Shirakawa, Yasuo Mori, Takayuki Nakagawa, Shuji Kaneko

**Affiliations:** ^1^Department of Molecular Pharmacology, Graduate School of Pharmaceutical Sciences, Kyoto University, Kyoto, Japan; ^2^Department of Synthetic Chemistry and Biological Chemistry, Graduate School of Engineering, Kyoto University, Kyoto, Japan; ^3^Department of Physiology, Graduate School of Medical Sciences, Fukuoka University, Fukuoka, Japan; ^4^Department of Clinical Pharmacology and Therapeutics, Kyoto University Hospital, Kyoto, Japan

**Keywords:** TRPA1, oxaliplatin, reactive oxygen species, cold hypersensitivity, prolyl hydroxylase, cysteine oxidation, peripheral neuropathy

## Abstract

Oxaliplatin, a third-generation platinum-based chemotherapeutic agent, displays unique acute peripheral neuropathy triggered or enhanced by cold, and accumulating evidence suggests that transient receptor potential ankyrin 1 (TRPA1) is responsible. TRPA1 is activated by oxaliplatin via a glutathione-sensitive mechanism. However, oxaliplatin interrupts hydroxylation of a proline residue located in the N-terminal region of TRPA1 via inhibition of prolyl hydroxylase (PHD), which causes sensitization of TRPA1 to reactive oxygen species (ROS). Furthermore, PHD inhibition endows cold-insensitive human TRPA1 (hTRPA1) with ROS-dependent cold sensitivity. Since cysteine oxidation and proline hydroxylation regulate its activity, their association with oxaliplatin-induced TRPA1 activation and acquirement of cold sensitivity were investigated in the present study. A high concentration of oxaliplatin (1 mM) induced outward-rectifier whole-cell currents and increased the intracellular Ca^2+^ concentration in hTRPA1-expressing HEK293 cells, but did not increase the probability of hTRPA1 channel opening in the inside-out configuration. Oxaliplatin also induced the rapid generation of hydrogen peroxide, and the resultant Ca^2+^ influx was prevented in the presence of glutathione and in cysteine-mutated hTRPA1 (Cys641Ser)-expressing cells, whereas proline-mutated hTRPA1 (Pro394Ala)-expressing cells showed similar whole-cell currents and Ca^2+^ influx. By contrast, a lower concentration of oxaliplatin (100 μM) did not increase the intracellular Ca^2+^ concentration but did confer cold sensitivity on hTRPA1-expressing cells, and this was inhibited by PHD2 co-overexpression. Cold sensitivity was abolished by the mitochondria-targeting ROS scavenger mitoTEMPO and was minimal in cysteine-mutated hTRPA1 (Cys641Ser or Cys665Ser)-expressing cells. Thus, high oxaliplatin evokes ROS-mediated cysteine oxidation-dependent hTRPA1 activation independent of PHD activity, while a lower concentration induces cold-induced cysteine oxidation-dependent opening of hTRPA1 via PHD inhibition.

## Introduction

Oxaliplatin (L-OHP), a third-generation platinum-based agent, is frequently used to treat locally advanced and metastatic cancers of the colon or rectum. However, it increases the incidence of chemotherapy-induced peripheral neuropathy (CIPN), often resulting in chemotherapeutic dose delay or treatment discontinuation (Falcone et al., 2007; Miltenburg and Boogerd, 2014). In addition to cumulative and chronic CIPN after multiple chemotherapy cycles, oxaliplatin induces a peculiar acute CIPN, characterized by paresthesia, dysesthesia, or acral numbness, in ~90% of patients during or within hours of infusion. Acute CIPN is specific to oxaliplatin and often triggered or exacerbated by cold exposure (Wilson et al., 2002; Miltenburg and Boogerd, 2014; Cavaletti and Marmiroli, 2015).

The mechanisms underlying L-OHP-induced chronic CIPN can be explained, at least in part, by neurotoxicity in peripheral sensory neurons due to mitochondrial dysfunction and generation of reactive oxygen species (ROS) (Joseph and Levine, 2009; Di Cesare Mannelli et al., 2012; Azevedo et al., 2013) following accumulation of platinum in the dorsal root ganglia (DRG) (Screnci et al., 2000; Cavaletti et al., 2001). By contrast, L-OHP-induced acute CIPN is recognized as a channelopathy. A body of evidence suggests that it is caused by alteration of the kinetics of the axonal voltage-gated Na^+^ channel (Sittl et al., 2012; Deuis et al., 2013) and/or activation of transient receptor potential ankyrin 1 (TRPA1) (Nassini et al., 2011; Zhao et al., 2012).

TRPA1 is a polymodal cation channel that plays a pivotal role as a nociceptor (Wu et al., 2010; Viana, 2016). This channel is opened by a large number of irritant chemicals (Bandell et al., 2004; Jordt et al., 2004). TRPA1 is also activated by oxidative stimuli such as, ROS and hyperoxia (Takahashi et al., 2008, 2011). TRPA1 activation evoked by most irritant chemicals and oxidative stimuli is caused by covalent or oxidative modification of cysteine residues in the N-terminal region (Hinman et al., 2006; Macpherson et al., 2007). On the other hand, we previously identified another mechanism for TRPA1 activation; a decrease in oxygen concentration diminishes the activity of prolyl hydroxylases (PHDs) and relieves TRPA1 from the PHD-dependent hydroxylation of a proline residue (Pro^394^) located within the N-terminal ankyrin repeat domain, leading to hypoxia-induced activation (Takahashi et al., 2011; So et al., 2016).

It is reported that both L-OHP and cisplatin activate TRPA1 via glutathione-sensitive mechanisms (Nassini et al., 2011). However, we previously demonstrated that L-OHP and its characteristic metabolite oxalate enhance the responsiveness of TRPA1, which may contribute to the cold hypersensitivity induced by L-OHP in mice, but this is not the case for cisplatin and paclitaxel (Zhao et al., 2012). We also demonstrated the molecular mechanism: L-OHP and oxalate inhibit PHD activity, which augments the sensitivity of human TRPA1 (hTRPA1) to ROS by inhibiting hydroxylation of Pro^394^. Furthermore, we found that use of a PHD inhibitor or a hTRPA1 mutant lacking the hydroxylation-susceptible Pro^394^ residue induces hTRPA1 sensitization to ROS, which enables cold-insensitive hTRPA1 to sense cold by detecting cold-evoked ROS production (Miyake et al., 2016). Therefore, the cold-induced indirect activation of hTRPA1 that is sensitized by PHD inhibition may be responsible for L-OHP-induced acute CIPN triggered by cold, although whether L-OHP actually endows cold sensitivity to hTRPA1-expressing cells has not been clarified. Thus, L-OHP is likely to activate and sensitize TRPA1, but whether the L-OHP-induced TRPA1 activation and sensitization is due to the oxidation of cysteine residues and/or inhibition of proline hydroxylation remains unknown.

In this study, we investigated whether and how cysteine oxidation and/or inhibition of proline hydroxylation contribute to the L-OHP-induced hTRPA1 activation and sensitization *in vitro*. A high concentration of L-OHP evoked cysteine oxidation-dependent hTRPA1 activation, independent of hydroxylation of the PHD-targeted proline residue, while a subthreshold concentration of L-OHP endowed hTRPA1 with cysteine oxidation-dependent cold sensitivity through PHD inhibition.

## Materials and methods

### Reagents

L-OHP and allyl isothiocyanate (AITC) were purchased from Wako Pure Chemical Industries (Osaka, Japan). N-tert-butyl-α-phenylnitrone (PBN), cremophore EL, 2-aminoethoxydiphenyl borate (2-APB), poly-L-lysine, and D-mannitol were purchased from Sigma-Aldrich (St. Louis, MO). The 1,2-bis(2-aminophenoxy)ethane-N,N,N′,N′-tetraacetic acid (BAPTA) was acquired from Dojindo Laboratories (Kumamoto, Japan). The mitoTEMPO was obtained from Santa Cruz (Dallas, TX). Peroxy Green 1 (PG-1) was synthesized previously (Miyake et al., 2016) according to the literature (Miller et al., 2007). Other drugs and chemicals were obtained from Nacalai Tesuque (Kyoto, Japan).

### Plasmids

Constructs consisting of recombinant hTRPA1, its cysteine mutants (C633S, C641S, C665S), its proline mutant (P394A), or the human PHD2 cDNA in the pCIneo expression vector were prepared previously (Takahashi et al., 2011). The pEGFP-C3 was purchased from Clontech Laboratories (Madison, WI).

### Cell cultures and transfection

HEK293 cells were cultured in Dulbecco's modified Eagle's medium (DMEM) with GlutaMAX I (10566-016, Life Technologies) supplemented with 10% heat-inactivated fetal bovine serum (Sigma) and maintained at 37°C in a humidified incubator set at 5% CO_2_. HEK293 cells were co-transfected with recombinant plasmids and pEGFP-C3 using SuperFect Transfection Reagent (Qiagen, Hilden, Germany) or Lipofectamine 2000 (Life Technologies). Two days after transfection, cells were placed onto coverslips coated with poly-L-lysine and used in electrophysiological recording or fluorometric imaging.

### Electrophysiology

Electrophysiological recordings were performed with a pipette made from a glass capillary (outer diameter, 1.5 mm) with an internal filament (Narishige, Tokyo, Japan) pulled using a P-87 micropipette puller (Sutter, Novato, CA). Access resistance ranged from 2 to 5 MΩ when the pipette was filled with pipette solution described below. For whole-cell patch-clamp recordings, the bath solution contained 100 mM NaCl, 2 mM CaCl_2_, and 10 mM HEPES (adjusted to pH 7.4 with NaOH and 300 mOsm with D-mannitol), and the pipette solution contained 100 mM Cs-aspartate, 5 mM BAPTA, 1.4 mM Ca-gluconate (30 nM free Ca^2+^), 2 mM MgSO_4_, 2 mM MgCl_2_, 4 mM Na_2_-ATP, 10 mM Na_5_P_3_O_10_, and 10 mM HEPES (adjusted to pH 7.4 with CsOH and 300 mOsm with D-mannitol). Current-voltage relationships were measured using voltage ramps (−100 to +100 mV over 100 ms) applied every 10 s. The membrane potential was set at 0 mV. Access resistance values were compensated by 70%. For inside-out patch-clamp recordings, the bath solution contained 50 mM Cs-aspartate, 50 mM CsCl, 10 mM EGTA, 1 mM CaCl_2_ (10 nM free Ca^2+^), 1 mM MgCl_2_, 10 mM Na_5_P_3_O_10_, and 10 mM HEPES (adjusted to pH 7.4 with CsOH and 300 mOsm with D-mannitol), and the pipette solution contained 100 mM CsCl, 1 mM MgCl_2_, 1 mM EGTA, and 10 mM HEPES (adjusted to pH 7.4 with CsOH and 300 mOsm with D-mannitol). The membrane potential was set at +80 mV. Data were filtered at 2.9 kHz. Experiments were conducted at room temperature. Patch-clamp recordings were performed using an EPC-10 patch-clamp amplifier (HEKA Instruments, Lambrecht, Germany) and PATCHMASTER software (HEKA). AITC (100 μM) was used to validate the expression of hTRPA1. The representative trace was obtained at least three independent experiments.

### Measurement of intracellular Ca^2+^ concentration ([Ca^2+^]_i_)

Cells on coverslips were loaded for 30–40 min with 5 μM Fura-2 acetoxymethyl ester (Fura-2 AM; Dojindo Laboratories) in Krebs-Ringer solution containing 140 mM NaCl, 5 mM KCl, 1 mM MgCl_2_, 2 mM CaCl_2_, 10 mM glucose, and 10 mM HEPES (pH adjusted to 7.4 with NaOH) containing 0.005% cremophore EL. Fluorescence images were captured every 5 s using alternating excitation at 340 and 380 nm and emission at 510 nm with an AQUACOSMOS/ORCA-AG imaging system (Hamamatsu Photonics, Shizuoka, Japan). For the pretreatment, L-OHP (100 μM) was added to the culture medium 2 h before loading. MitoTEMPO (10 μM, 10 mM stock solution in DMSO was diluted with the Fura-2 contained Krebs-Ringer solution) was preloaded with Fura-2 loading. Note that all drugs used for pretreatments were removed by washing before Ca^2+^ imaging experiments. Experiments were conducted at room temperature unless otherwise stated. Cold stimulation was performed with an SC-20 dual in-line solution heater/cooler and a CL-100 temperature controller (Warner Instruments, Hamden, CT). The velocity of the cooling ramp is about 3.75°C/min. The ratio of the fluorescence intensity obtained by excitation/emission at 340 nm/510 nm (F_340_) to the fluorescence intensity obtained by excitation/emission at 380 nm/510 nm (F_380_), namely, F_340_/F_380_, was calculated to quantify the intracellular Ca^2+^ concentration ([Ca^2+^]_i_). Cells with an F_340_/F_380_ ratio >1.5 at baseline were excluded. Statistical analysis of the change in the ratio, ΔRatio (F_340_/F_380_), was performed as follows; in **Figure 3**, the average ΔRatio (F_340_/F_380_) during 0–2 min after 2-APB-application was used; in **Figure 5**, the ΔRatio (F_340_/F_380_) at 2 min after cold stimulation was used. AITC (100 μM) or 2-APB (100 μM) was used to validate the expression of hTRPA1.

### Measurement of intracellular H_2_O_2_ level

Intracellular H_2_O_2_ level was measured using PG-1, a fluorescent probe with high selectivity for H_2_O_2_ (Miller et al., 2007). Cells on coverslips were loaded for 30–40 min with 5 μM PG-1 in HEPES-buffered saline containing 107 mM NaCl, 6 mM KCl, 1.2 mM MgSO_4_, 2 mM CaCl_2_, 11.5 mM D-glucose, and 20 mM HEPES (pH adjusted to 7.4 with NaOH). Fluorescence images were captured every 20 s using alternating excitation at 488 nm and emission at 510 nm with the AQUACOSMOS/ORCA-AG imaging system (Hamamatsu Photonics). Experiments were conducted at room temperature. The fluorescence intensity obtained with excitation/emission of 488 nm/510 nm relative to the values obtained at 0 min (F/F_0_) was calculated to validate the intracellular H_2_O_2_ concentration. The ΔF/F_0_ obtained from each cells at 15 min was used for statistical analysis.

### Statistical analysis

**Figure 1:**
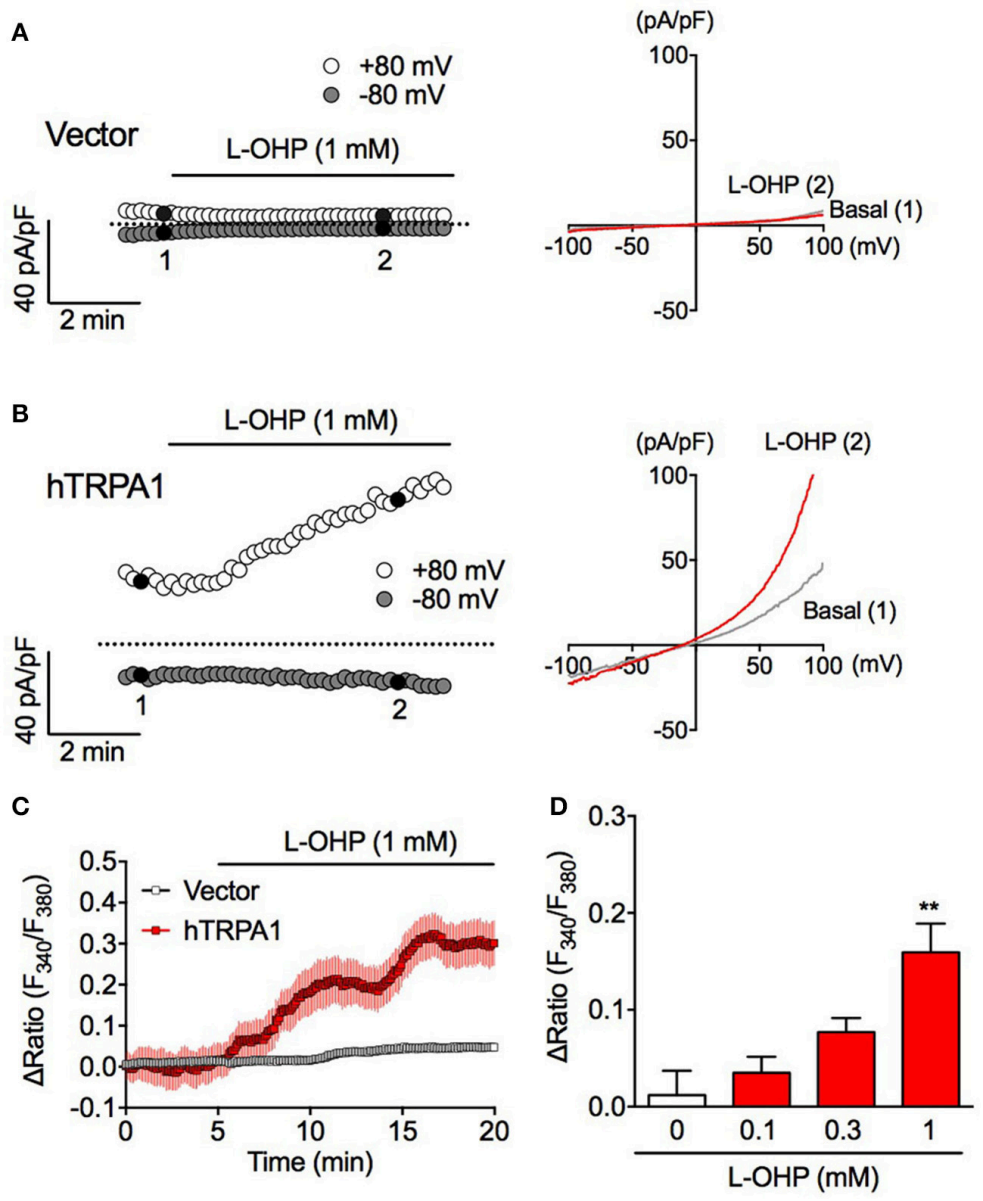
High concentration of L-OHP induces hTRPA1 activation. **(A,B)** Effect of L-OHP (1 mM) on the whole-cell currents in HEK293 cells transfected with control **(A)** or hTRPA1 **(B)** vector. Left traces are representative whole-cell recordings. The current-voltage relationships were acquired at the time indicated by black-filled circle in **(A,B)**, respectively. Membrane potential was set at 0 mV. **(C)** Effect of L-OHP on the intracellular Ca^2+^ concentration in HEK293 cells transfected with control or hTRPA1 vector (*n* = 26–53 cells). Left panel shows representative traces of intracellular Ca^2+^ imaging. Panel **(D)** shows the statistical analysis for concentration-dependent effect of L-OHP (0.1, 0.3, and 1 mM). *n* = 4–8 independent experiments. ^**^P < 0.01 vs. the vehicle-treated hTRPA1-expressing cells (Veh). Panels **(C,D)** are expressed as mean ± S.E.M. All of the experiments were performed at room temperature.

**Figure 2:**
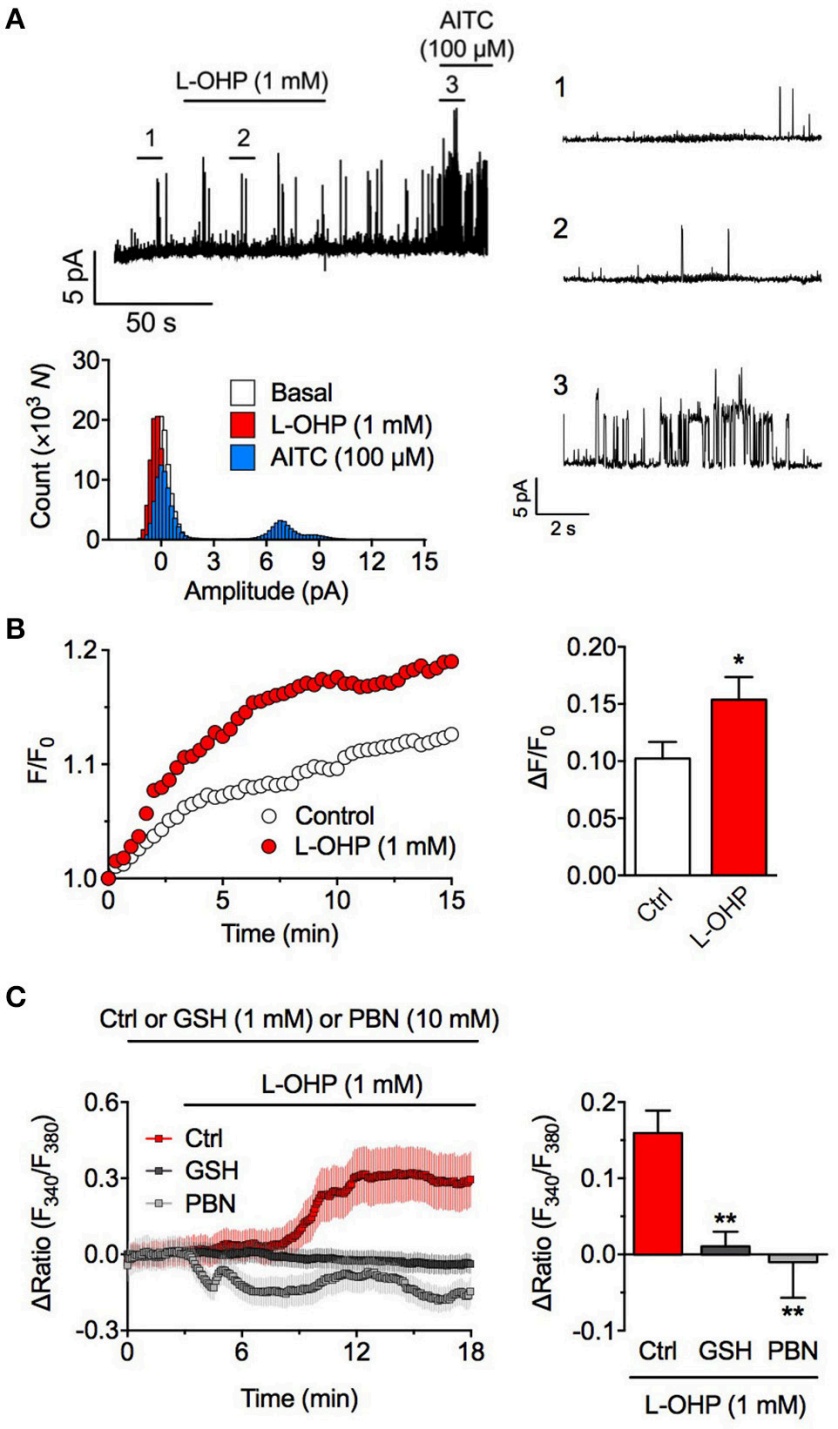
High concentration of L-OHP activates hTRPA1 via ROS generation. **(A)** Effect of L-OHP (1 mM) on the open probability of hTRPA1 in inside-out recordings from hTRPA1-expressing HEK293 cells. Left upper panel shows a representative trace, right panels show the magnified traces at the time point as indicated in the left trace, and left lower panel shows their histogram. Membrane potential was set at +80 mV. Note that even at positive voltage, weak voltage-dependent TRPA1 was not activated by L-OHP. **(B)** Effect of L-OHP (1 mM) on the intracellular H_2_O_2_ concentration in HEK293 cells. Left panel shows representative traces of intracellular H_2_O_2_ imaging using PG-1, and right panel shows its statistical analysis. *n* = 28–32 cells from two independent experiments. ^*^P < 0.05 vs. vehicle-treated hTRPA1-expressing cells (Ctrl). **(C)** Effect of pretreatment with glutathione (1 mM) or PBN (10 mM) on the L-OHP-evoked [Ca^2+^]_i_ increase in hTRPA1-expressing HEK293 cells. Left panel shows representative traces of intracellular Ca^2+^ imaging (*n* = 22–34 cells), and right panel shows its statistical analysis (*n* = 5–8 independent experiments). ^**^P < 0.01 vs. non-treated hTRPA1-expressing cells (Ctrl). All of the experiments were performed at room temperature. All data except for **(A)** are expressed as mean ± S.E.M. GSH; glutathione.

**Figure 3:**
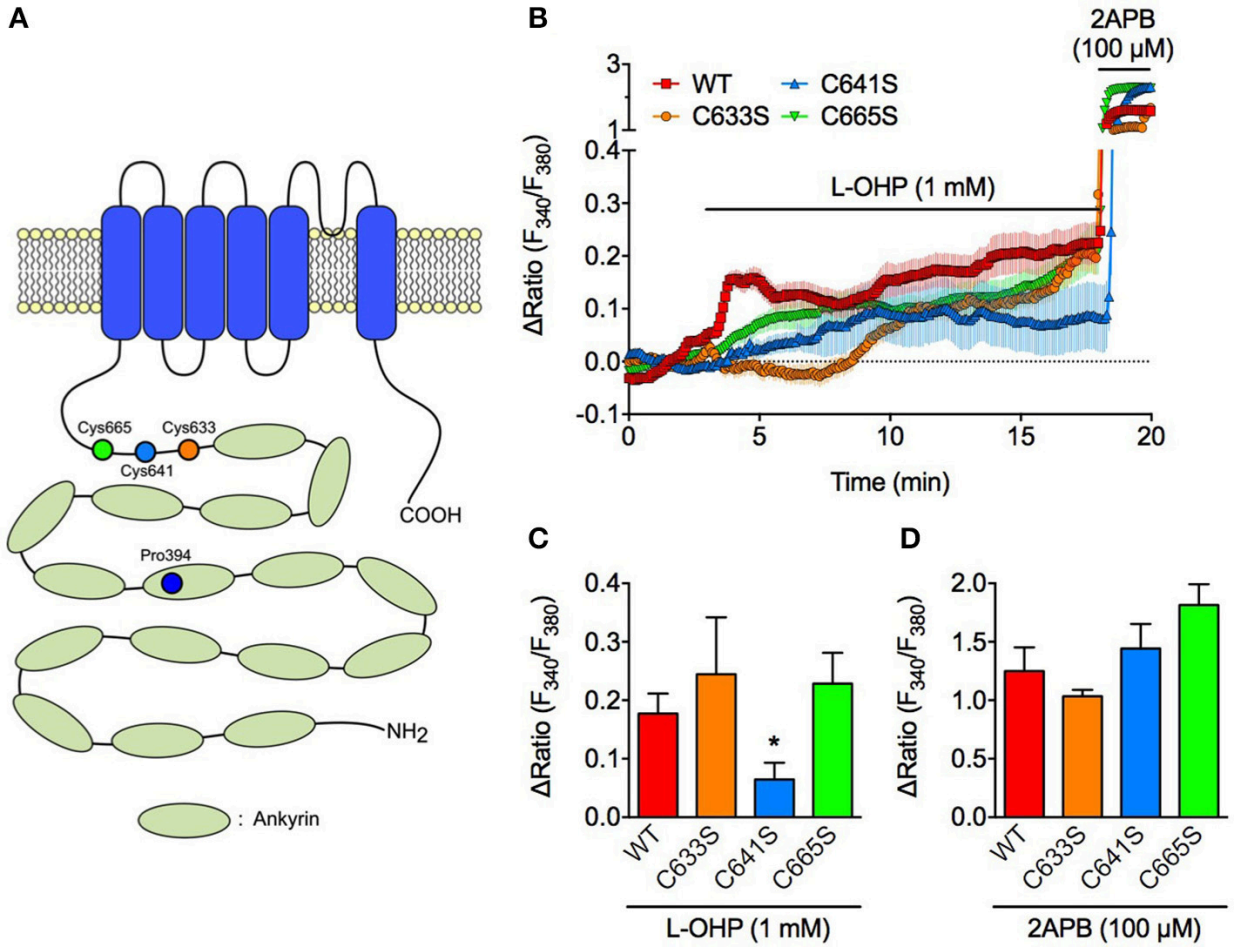
High concentration of L-OHP activates hTRPA1 via Cys641 modification. Effect of L-OHP (1 mM) on the intracellular Ca^2+^ concentration in HEK293 cells expressing hTRPA1 cysteine mutants (hTRPA1-C633S, C641S, and C665S). 2-APB (100 μM) was used to validate the expression of hTRPA1 cysteine mutants. **(A)** Schematic diagram illustrates the location of cysteine and proline residues of hTRPA1 that we focused in this study. **(B–D)** Representative traces of intracellular Ca^2+^ imaging (*n* = 42–92 cells) **(B)** and its statistical analysis for L-OHP- and 2-APB-evoked [Ca^2+^]_i_ increase **(C,D)** (*n* = 5–10 independent experiments). Note that we used 2-APB instead of AITC, since AITC activates TRPA1 via Cys oxidation and not suitable for examining the full activation of TRPA1 in **(D)**. ^*^P < 0.05 vs. wildtype hTRPA1-expressing cells (WT). All of the experiments were performed at room temperature. All data are expressed as mean ± S.E.M.

The data are presented as means ± S.E.M. from *n* independent experiments or cells. Statistical significances were calculated using GraphPad Prism 7 (GraphPad Software, La Jolla, CA). The data in **Figures 2B,C**, **3C,D**, **5B,C** were compared using unpaired Student's *t*-tests (vs. Ctrl in **Figure 2C**, vs. WT in **Figures 3C,D**, **5C**). The data in Figures 1D, **5A** were compared using one-way analyses of variance (ANOVA), followed by Tukey's multiple comparisons test. In all cases, *P* < 0.05 were considered statistically significant.

## Results

### A high concentration of L-OHP induces hTRPA1 activation

To investigate whether L-OHP activates hTRPA1, we performed whole-cell patch-clamp recording and fura-2-based intracellular Ca^2+^ imaging experiments. In vector-transfected HEK293 cells, L-OHP (1 mM) did not induce a membrane current (Figure 1A). Meanwhile, in hTRPA1-expressing cells, L-OHP (1 mM) induced gradually increasing TRPA1-like outward-rectifier currents (Figure 1B). Intracellular Ca^2+^ imaging revealed that L-OHP (1 mM) also significantly increased [Ca^2+^]_i_ in hTRPA1-expressing cells in a concentration-dependent manner (Figure 1C). However, no statistically significant increase was observed at lower concentrations of L-OHP (0.1 or 0.3 mM). These results indicate that L-OHP activates hTRPA1 when present at a concentration of at least 1 mM.

### High L-OHP-induced ROS generation is required for hTRPA1 activation

To clarify whether L-OHP activates hTRPA1 directly, we performed inside-out patch-clamp recording experiments for removing intracellular components. We held the membrane potential at +80 mV, at which point weak voltage-gated hTRPA1 is easy to open. Although AITC (100 μM) increased the probability of hTRPA1 opening, a high concentration of L-OHP (1 mM) had no effect (Figure 2A), suggesting that high L-OHP activates hTRPA1 indirectly. Since L-OHP reportedly induces mitochondrial dysfunction and triggers ROS generation (Zheng et al., 2011), we examined the effect of high L-OHP (1 mM) on the intracellular ROS level using the H_2_O_2_-specific indicator PG-1 (Miller et al., 2007). In HEK293 cells, high L-OHP induced H_2_O_2_ generation within 15 min (Figure 2B). Furthermore, the high L-OHP-evoked [Ca^2+^]_i_ increase was significantly suppressed in the presence of the antioxidants glutathione (1 mM) or PBN (10 mM) (Figure 2C). These results suggest that high L-OHP does not directly activate hTRPA1 but rather triggers ROS generation, which causes glutathione-sensitive hTRPA1 activation.

### High L-OHP-evoked hTRPA1 activation is regulated by cysteine oxidation and is independent of PHDs

Since ROS-induced TRPA1 activation is caused by oxidative modulation of the cysteine residues in the N-terminal region of TRPA1 (Takahashi et al., 2008; Figure 3A), we compared the high L-OHP-evoked [Ca^2+^]_i_ increase in HEK293 cells expressing wild-type hTRPA1 (hTRPA1-WT) or hTRPA1 cysteine mutants (hTRPA1-C633S, C641S, C665S) in which each ROS or oxygen-sensitive cysteine residue was replaced with serine, which is a well-characterized strategy to investigate the redox sensitivity of cysteine residues in TRPA1 (Macpherson et al., 2007; Takahashi et al., 2008, 2011). Among the hTRPA1 cysteine mutants, hTRPA1-C641S showed a significantly weaker response to high L-OHP (Figures 3B,C), while the responses of the other mutants (C633S and C665S) were comparable with hTRPA1-WT. By contrast, the response to 2-APB (100 μM), a cysteine oxidation-independent TRPA1 agonist (Hinman et al., 2006; Hu et al., 2009b), did not differ between hTRPA1-WT and its mutants (Figures 3B,D).

**Figure 4:**
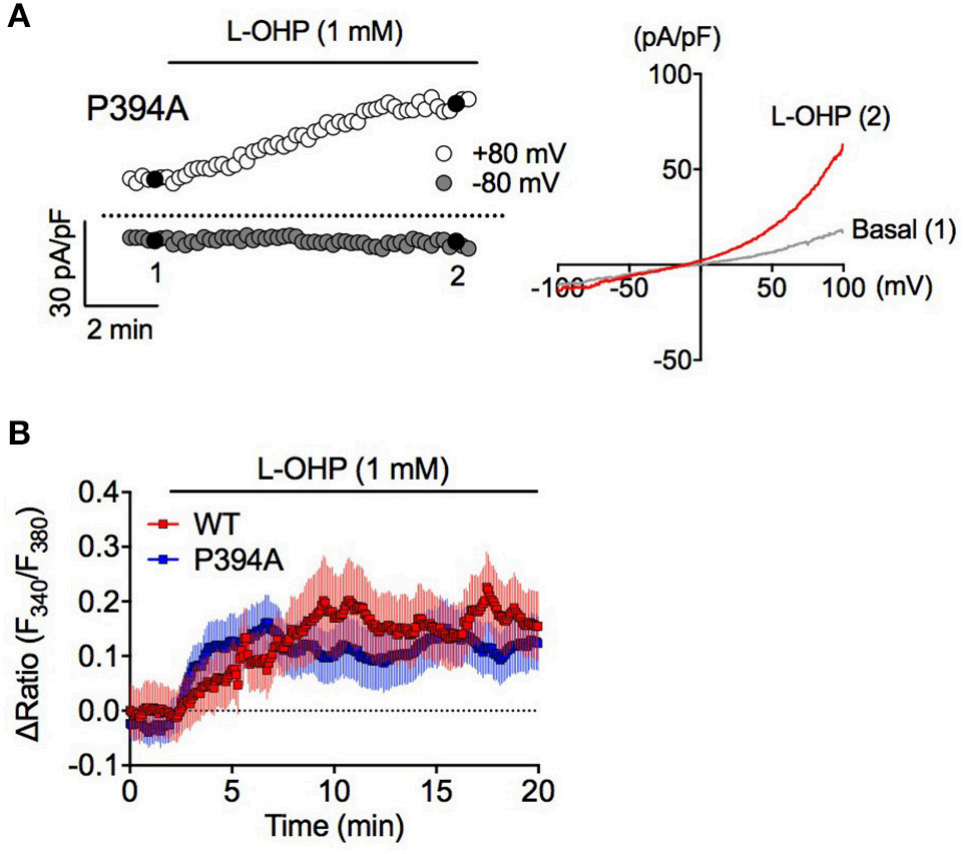
High L-OHP evoked hTRPA1 activation is independent of PHD modification. **(A)** Effect of L-OHP (1 mM) on the whole-cell currents in HEK293 cells transfected with hTRPA1-P394A mutant vector. Left traces are representative whole-cell recordings. The current-voltage relationships were acquired at the time indicated by black-filled circle, respectively. Membrane potential was set at 0 mV. **(B)** Representative traces of intracellular Ca^2+^ imaging of HEK293 cells transfected with hTRPA1-WT or hTRPA1-P394A mutant vector (*n* = 26–50 cells). Panel **(B)** is expressed as mean ± S.E.M. All of the experiments were performed at room temperature.

To investigate whether PHD inhibition is involved in high L-OHP-evoked hTRPA1 activation, we examined whether high L-OHP activates a PHD inhibition-insensitive hTRPA1 mutant hTRPA1-P394A. In whole-cell patch-clamp recordings, L-OHP (1 mM) successfully induced TRPA1-like outward-rectifier currents in hTRPA1-P394A expressing cells (Figure 4A). In Ca^2+^ imaging experiments, both hTRPA1-WT and hTRPA1-P394A showed a [Ca^2+^]_i_ increase induced by high L-OHP, and we did not observed any difference between them [Figure 4B, *n* = 6 independent experiments, *P* = 0.233 vs. hTRPA1-WT (Figure 1D, 1 mM L-OHP treated group, *n* = 8 independent experiments)]. These results suggest that high L-OHP activates hTRPA1 in a cysteine oxidation-dependent manner, while L-OHP-induced PHD inhibition is not involved in this phenomenon.

### A low concentration of L-OHP endows cold sensitivity of hTRPA1 via both PHD inhibition and cysteine oxidation

**Figure 5:**
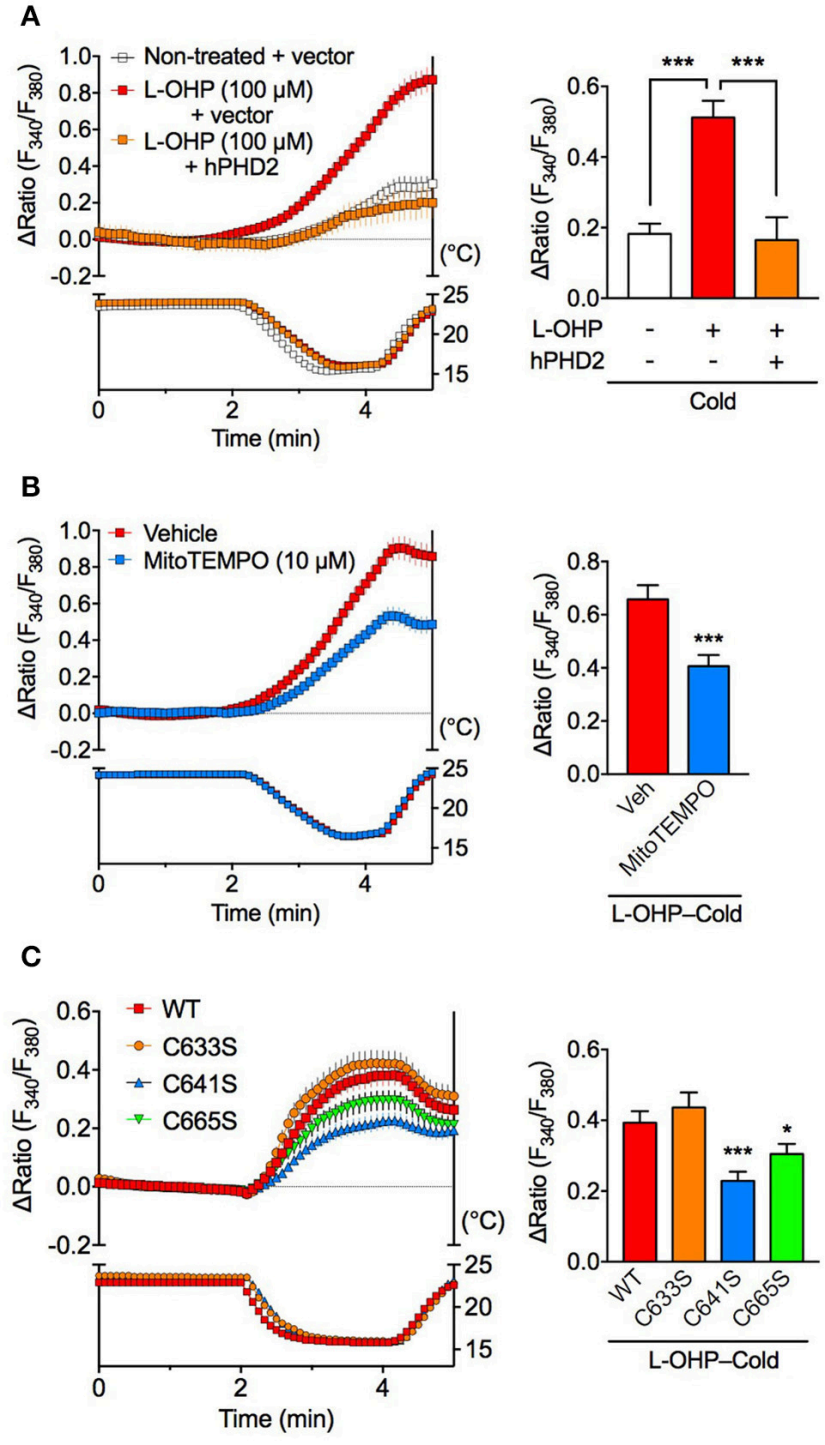
Cold-induced activation of hTRPA1 sensitized by low L-OHP is dependent on PHD inhibition and cysteine oxidation. Effects of PHD2 overexpression **(A)**, a mitochondria-targeted ROS scavenger **(B)** or hTRPA1 cysteine mutants **(C)** on the cold-evoked [Ca^2+^]_i_ increase following pretreatment with L-OHP (100 μM) for 2 h in hTRPA1-expressing HEK293 cells were investigated. **(A)** Left panel shows representative traces of intracellular Ca^2+^ imaging from hTRPA1-expressing HEK293 cells co-transfected with or without human PHD2 (top) and the temperature of the recording solution (bottom). Right panel shows its statistical analysis (*n* = 47–83 cells from two independent experiments). ^***^P < 0.001. **(B)** Left panel shows representative traces of intracellular Ca^2+^ imaging from L-OHP-treated hTRPA1-expressing HEK293 cells pretreated with vehicle or mitoTEMPO (10 μM, loading with Fura-2 Ca^2+^ indicator; top) and the temperature of the recording solution (bottom). Right panel shows its statistical analysis (*n* = 94–95 cells from two independent experiments). ^***^P < 0.001 vs. vehicle (0.1% DMSO)-treated cells (Veh). **(C)** Left panel shows representative traces of intracellular Ca^2+^ imaging from L-OHP-treated HEK293 cells expressing wildtype hTRPA1 (WT) or hTRPA1 cysteine mutants (hTRPA1-C633S, C641S, and C665S; top) and the temperature of the recording solution (bottom). Right panel shows its statistical analysis (*n* = 202–266 cells from three independent experiments). ^*^P < 0.05, ^***^P < 0.001 vs. WT. All data are expressed as mean ± S.E.M.

We recently reported that PHD inhibition causes hTRPA1 sensitization to ROS, which allows hTRPA1 to sense cold indirectly via cold-induced ROS generation (Miyake et al., 2016). Consistently, cold stimulation had minimal effect on [Ca^2+^]_i_ in non-treated hTRPA1-expressing cells in the present study, while pretreatment with a relatively low concentration of L-OHP (100 μM) for 2 h significantly increased [Ca^2+^]_i_ compared with that in non-treated hTRPA1-expressing cells. The cold-evoked [Ca^2+^]_i_ increase following low L-OHP pretreatment was partially but significantly inhibited abolished in cells co-expressing hTRPA1 and PHD2 (Figure 5A) and in hTRPA1-expressing cells pretreated with the mitochondria-targeting ROS scavenger mitoTEMPO (10 μM; Figure 5B). To investigate whether hTRPA1 cysteine residues are involved in the low L-OHP-endowed cold-evoked [Ca^2+^]_i_ increase, we performed the same experiments using hTRPA1 cysteine mutants. The cold-evoked [Ca^2+^]_i_ increase following low L-OHP pretreatment was significantly smaller in hTRPA1-C641S and hTRPA1-C665S mutants than that in hTRPA1-WT, while there was no difference between hTRPA1-WT and the hTRPA1-C633S mutant (Figure 5C). These results suggest that the low L-OHP-endowed cold sensitivity of hTRPA1 is dependent on both PHD inhibition and cysteine oxidation, and cold-induced mitochondrial ROS generation is important for the cold-evoked activation of hTRPA1 sensitized by low L-OHP.

## Discussion

**Figure 6:**
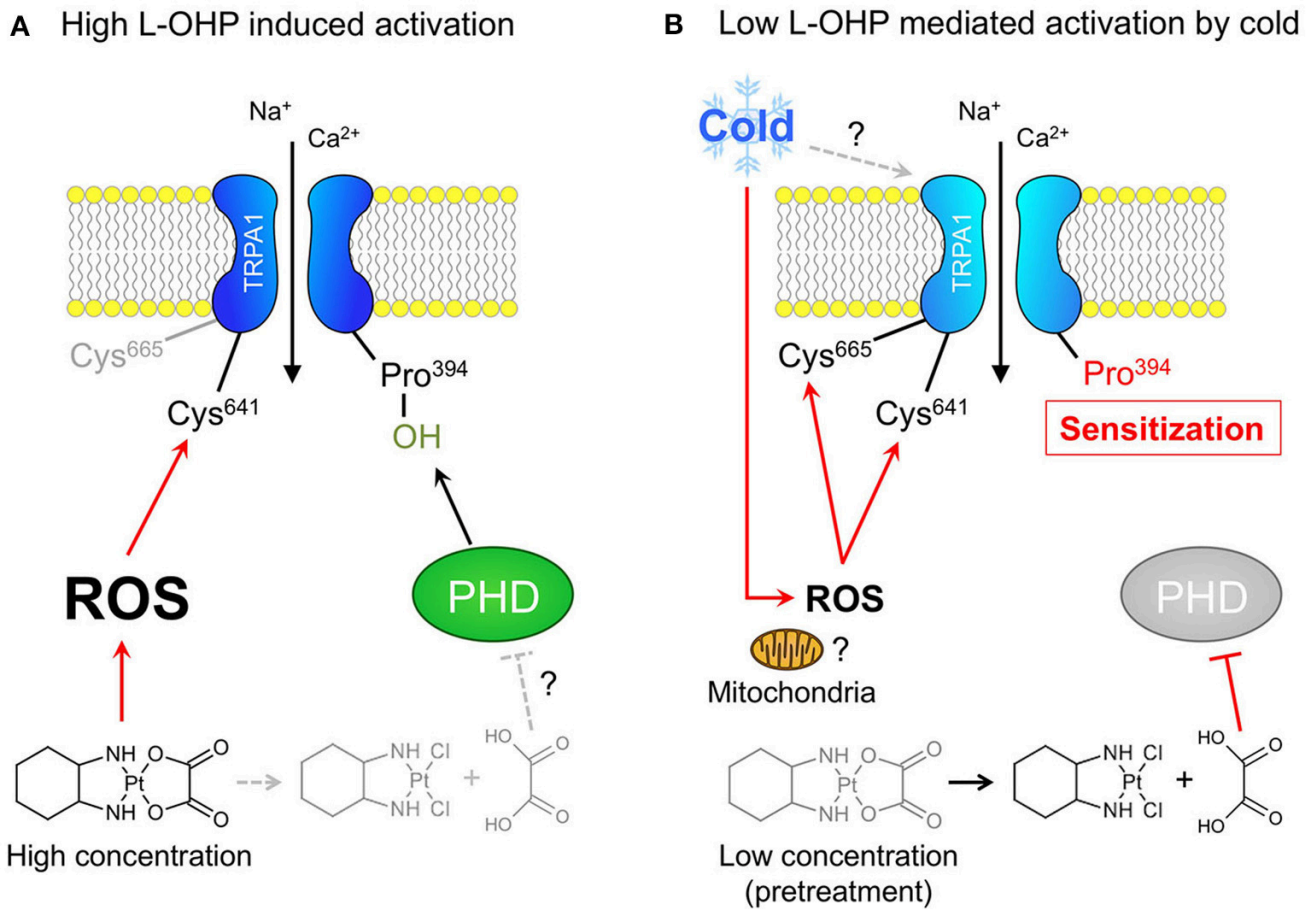
Different concentration of L-OHP elicits cysteine oxidation-dependent activation or cold sensitization of hTRPA1. **(A)** The mechanism underlying the high L-OHP-induced hTRPA1 activation. L-OHP induces ROS generation (presumably via mitochondria), and the resultant ROS activate hTRPA1 via oxidation of Cys641. **(B)** The mechanism underlying the low L-OHP-mediated hTRPA1 activation by cold. The long exposure of L-OHP (more than 1 h) causes the inhibition of PHDs by oxalate, a metabolite of L-OHP, which results in the sensitization of hTRPA1. The sensitized hTRPA1 is activated by ROS produced by mitochondria following cold exposure via oxidation of Cys 641 and Cys665.

Among others, TRPA1 is activated through dual mechanisms: covalent or oxidative modification of cysteine residues, and inhibition of hydroxylation of a proline residue in the N-terminal region (Takahashi et al., 2011). The results of the present study showed that a high concentration of L-OHP (≥1 mM) evoked hTRPA1 activation via ROS-mediated cysteine oxidation, independently of PHD inhibition, while both mechanisms are responsible for the cold-induced activation of hTRPA1 sensitized by the low concentration of L-OHP. The high concentration of L-OHP activated hTRPA1 in whole-cell patch clamp recordings and intracellular Ca^2+^ imaging experiments, but not membrane-excised inside-out patch clamp recordings, indicating that the high concentration of L-OHP affects cellular components other than hTRPA1 itself, and indirectly activates hTRPA1. Furthermore, glutathione and PBN suppressed the L-OHP (1 mM)-induced activation of hTRPA1, suggesting that the high concentration of L-OHP induces ROS production that is followed by the activation of hTRPA1 (Figure 6). We previously reported that the low concentration of L-OHP increases the sensitivity of hTRPA1 to ROS via PHD inhibition (Miyake et al., 2016). In this study, we further found that the L-OHP (100 μM)-pretreated hTRPA1-expressing cells showed larger response to cold compared with the control hTRPA1-expressing cells, which was not observed when we used PHD2-overexpressing cells, and was partially inhibited by the pretreatment with mitoTEMPO. The L-OHP dependent cold response was suppressed in ROS-sensitive cysteine-mutated hTRPA1-expressing cells, indicating that the pretreatment of L-OHP (100 μM) allows hTRPA1 to sense cold in the same mechanisms we revealed before (Miyake et al., 2016) via modification to cysteine residues (Figure 6).

TRPA1 can be activated by heavy metals such as, gold, zinc, and cadmium via cysteine modification (Hu et al., 2009a; Gu and Lin, 2010; Hatano et al., 2013; Miura et al., 2013). However, the concentration of the platinum compound L-OHP required for hTRPA1 activation (1 mM) is much higher than that reported previously for other heavy metals (μM) (Hu et al., 2009a; Hatano et al., 2013; Miura et al., 2013). The possibility that L-OHP directly activates hTRPA1 was ruled out since high L-OHP was unable to evoke TRPA1 activation in inside-out patch-clamp recording experiments. Rather, the results indicated indirect activation of hTRPA1 through L-OHP-induced ROS generation since (1) we detected an increase in intracellular H_2_O_2_ production following high L-OHP treatment, and (2) high L-OHP-evoked hTRPA1 activation was blocked by the membrane-impermeable antioxidant glutathione, consistent with a previous report (Nassini et al., 2011). It is reported that L-OHP induces apoptosis via mitochondrial damage (Gourdier et al., 2004), which triggers ROS generation (Bishop et al., 2010). Taken together, our findings suggest that high L-OHP triggers ROS generation, presumably by mitochondria, resulting in activation of TRPA1.

Cysteine oxidation is one of the most common mechanisms for TRPA1 activation by various agonists, and Cys633, Cys641, and Cys665 are crucial for activation by electrophiles (Hinman et al., 2006; Ibarra and Blair, 2013). Among these, Cys641 and Cys665 are important for activation by low concentrations of H_2_O_2_ (Takahashi et al., 2008) and nitric oxide (Kozai et al., 2014). Cys641 is also important for activation by zinc (Hu et al., 2009a) and cadmium (Miura et al., 2013), whereas Cys633 is involved in activation by HNO (Eberhardt et al., 2014) and the gold compound auranofin (Hatano et al., 2013). In the present study, we showed that mutation of Cys641 inhibited high L-OHP-evoked hTRPA1 activation. This result further supports indirect hTRPA1 activation by high L-OHP through ROS generation, although mutating Cys665 had no effect, which may partially contradict this finding. This paradox may indicate some additional roles of L-OHP in the high L-OHP-induced hTRPA1 activation, but further investigations are required. The fact that mutation of Cys633 did not affect high L-OHP-evoked hTRPA1 activation may suggest that platinum does not directly activate hTRPA1 in a cysteine-dependent manner, like gold.

Hydroxylation of the proline residue in the N-terminal region of TRPA1 by PHDs is critical for regulating TRPA1 activity (Miyake et al., 2016). Although L-OHP and its metabolite oxalate can inhibit PHDs (Miyake et al., 2016), the present results suggest that PHDs are not involved in the high L-OHP-evoked hTRPA1 activation. This apparent discrepancy may be explained by the previous observation that induction of TRPA1 sensitization in mouse DRG neurons by low L-OHP pretreatment requires more than 1 h (Zhao et al., 2012). Thus, it is likely that inhibition of PHDs and induction of TRPA1 sensitization by L-OHP and/or oxalate may be slower than the high L-OHP-induced rapid ROS generation that activates TRPA1. It is probable that cysteine oxidation by ROS, rather than inhibition of proline hydroxylation by L-OHP and/or oxalate and subsequent delayed sensitization of hTRPA1, contributes to hTRPA1 activation evoked by high L-OHP.

We previously reported that PHD inhibition by a PHD inhibitor dimethyloxalylglycine (DMOG) sensitizes hTRPA1 to ROS and induces channel opening at cold temperatures. Furthermore, similar cold hypersensitivity is also observed in the mice treated with DMOG, which is also inhibited by a TRPA1 antagonist HC030031 (Miyake et al., 2016). Consistently, in the present study, pretreatment with a relatively low concentration of L-OHP potentiated the cold sensitivity of hTRPA1. Furthermore, this L-OHP-induced cold sensitivity was significantly reduced when treated with a mitochondria-targeting ROS scavenger, suggesting that ROS generated from mitochondria during cold exposure contributes to the L-OHP-induced cold sensitivity, similar to DMOG. Consistent with our results, previous *in vivo* experiments showed that L-OHP-induced cold hypersensitivity was attenuated by a single acute administration of a ROS scavenger (Miyake et al., 2016) or a mitochondria-targeting ROS scavenger (Toyama et al., 2014). Furthermore, the results obtained from Cys641 and Cys665 hTRPA1 mutants confirmed that these residues are responsible for activation by H_2_O_2_ (Takahashi et al., 2008) and contribute to the indirect cold sensitivity of hTRPA1 induced by low L-OHP. These results suggest that ROS presumably generated from mitochondria during cold exposure oxidize cysteine residues in the N-terminal region of hTRPA1, thereby activating hTRPA1 following exposure to low L-OHP pretreatment. However, in this study, mitoTEMPO did not completely inhibit the cold-induced increase of [Ca^2+^]_i_ in the low L-OHP-treated hTRPA1 expressing cells. This mitoTEMPO-insensitive component may be ROS-independent but still PHD-dependent, since the over-expression of PHD2 completely inhibited the cold-induced hTRPA1 activation and hTRPA1-P394A mutant (that mimics a constitutively PHD-inhibited condition) shows a weak ROS-independent cold sensitivity (Miyake et al., 2016). By contrast, the mutation demonstrated that Cys633 had no effect on the indirect cold sensitivity of hTRPA1, which may indicate that platinum itself is not involved in this phenomenon. This hypothesis is consistent with our previous findings that other platinum-based chemotherapeutic agents such as, cisplatin and carboplatin do not induce acute cold hypersensitivity (Zhao et al., 2012).

The concentration of L-OHP in commercial infusions is about 1.25 mM, while the calculated blood concentration in patients is < 100 μM (Chalret du Rieu et al., 2014). Although L-OHP accumulates in the DRG and peripheral nerves (Screnci et al., 2000; Cavaletti et al., 2001), the concentration of L-OHP required to evoke TRPA1 activation (1 mM) appears to be too high to explain L-OHP-induced acute CIPN. In addition, a high concentration of cisplatin, which does not induce acute CIPN, also activates TRPA1 via ROS generation (Nassini et al., 2011). Thus, high L-OHP-evoked TRPA1 activation via ROS generation is unlikely to be responsible for acute CIPN following L-OHP treatment. Interestingly, delayed mechanical, thermal, and cold hypersensitivity following repeated administration of L-OHP in rodents is prevented by some antioxidants (Joseph and Levine, 2009; Di Cesare Mannelli et al., 2012; Azevedo et al., 2013) and a TRPA1 blocker (Nassini et al., 2011). Furthermore, TRPA1 activation via ROS is associated with mechanical hypersensitivity induced by cisplatin (Nassini et al., 2011) and some other classes of chemotherapeutic agents such as, paclitacel (Materazzi et al., 2012), bortezomib (Trevisan et al., 2013), and vincristine (Old et al., 2014). Thus, ROS-mediated TRPA1 activation may be a common mechanism for cumulative and chronic CIPN.

In conclusion, we further clarified the molecular details of how L-OHP activates or sensitizes hTRPA1. L-OHP exhibited complex concentration-dependent effects on hTRPA1; high L-OHP evoked hTRPA1 activation in a proline-independent manner, while low L-OHP sensitized hTRPA1 in a proline-dependent manner. This finding implies that the same chemical agent can function via different molecular mechanisms to regulate target proteins in a concentration-dependent manner. Nevertheless, the present results provide experimental evidence that TRPA1 blockage may be of clinical benefit for CIPN patients treated with L-OHP.

## Author contributions

TM, TNa, and SK designed the project. TM, SN, ZM, SH, and KI performed the experiments. TM, SN, ZM, SH, HS, KN, and TNa analyzed the data; TNu, NT, and YM provided materials and technical advices. TM, TNa, and SK wrote the manuscript. SK supervised the experiments and finalized the manuscript.

### Conflict of interest statement

The authors declare that the research was conducted in the absence of any commercial or financial relationships that could be construed as a potential conflict of interest.
